# Recent Advances in Catalysis Based on Transition Metals Supported on Zeolites

**DOI:** 10.3389/fchem.2021.716745

**Published:** 2021-08-09

**Authors:** Perla Sánchez-López, Yulia Kotolevich, Rosario I. Yocupicio-Gaxiola, Joel Antúnez-García, Ramesh Kumar Chowdari, Vitalii Petranovskii, Sergio Fuentes-Moyado

**Affiliations:** ^1^ Departamento de Nanocatálisis, Centro de Nanociencias y Nanotecnología, Universidad Nacional Autónoma de México, Ensenada, Mexico; ^2^ Centro de Investigación Científica y de Educación Superior de Ensenada (CICESE), Ensenada, Mexico

**Keywords:** transition metals, zeolites, catalysts, enviromental protection, sustainable energy

## Abstract

This article reviews the current state and development of thermal catalytic processes using transition metals (TM) supported on zeolites (TM/Z), as well as the contribution of theoretical studies to understand the details of the catalytic processes. Structural features inherent to zeolites, and their corresponding properties such as ion exchange capacity, stable and very regular microporosity, the ability to create additional mesoporosity, as well as the potential chemical modification of their properties by isomorphic substitution of tetrahedral atoms in the crystal framework, make them unique catalyst carriers. New methods that modify zeolites, including sequential ion exchange, multiple isomorphic substitution, and the creation of hierarchically porous structures both during synthesis and in subsequent stages of post-synthetic processing, continue to be discovered. TM/Z catalysts can be applied to new processes such as CO_2_ capture/conversion, methane activation/conversion, selective catalytic NO_x_ reduction (SCR-deNO_x_), catalytic depolymerization, biomass conversion and H_2_ production/storage.

## Introduction

Zeolites^1^ are crystalline aluminosilicates with a negatively charged macromolecular inorganic framework, which display an extremely large surface area because of intracrystalline channels and cavities of molecular size with a characteristic geometry and architecture. They also contain freely moving and easily replaceable cations necessary to balance the charge of the Al containing tetrahedra (Breck, 1974).

Since the second half of the 20th century, zeolites have been widely used as catalysts for many reactions involving organic molecules such as cracking, isomerization, hydrocarbon synthesis, and selective oxidation (Gläser and Weitkamp, 2004; Yilmaz and Müller, 2009). Synthetic zeolites are catalysts of vital importance in petrochemical plants: they can be acid catalysts or can be used as support for active metals or reagents. Zeolites allow size- and shape-selectivity processes, either due to the discrimination of the transition state or to the exclusion of competing reagents depending on the molecule diameter, which allows for better control of the products.

The structural features and properties of zeolites make them one-of-a-kind unique catalyst carriers. In addition to the primary microporosity, post-synthetic treatment can create mesoporosity in their crystals, facilitating diffusion processes. The combination of micro- and mesoporosity creates new opportunities to develop chemical technology.

New challenges in chemistry and chemical technology, such as sustainability and green chemistry, have led to intensive development of synthesis and modification methods of zeolite catalysts (Li et al., 2017). Hierarchical zeolites are micro-mesoporous materials prepared based on standard zeolites, in which pores of different types and sizes are additionally generated. These pores can be intracrystalline, intercrystalline (between individual crystallites in agglomerated intergrowths), or inner space in materials such as layered pillared zeolites (Feliczak-Guzik, 2018). Pore-related characteristics, associated with the presence of pores of different diameters, determine specific applications of hierarchical zeolites, in particular, where diffusion within materials is of interest (Christensen et al., 2007; Pérez-Ramírez et al., 2008; Yocupicio-Gaxiola, et al., 2019).

New possibilities of using active phases containing ions and/or clusters of transition metals (TM) deposited on zeolites (TM/Z) are being investigated. The TM/Z can be prepared by ion exchange, incipient wetness impregnation, or deposition of metal complexes, followed by calcination or reduction to obtain oxidized states or metal nanoparticles. Hydrogen reduction of encaged transition metal ions can also yield metal clusters or isolated atoms in line with protons of high Brønsted acidity (Sachtler, 1992).

Most heterogeneous catalysts currently in practical use consist of one or more catalytically active compounds that are supported on carrier materials. The impregnation method is used to immobilize acids and bases, salts, oxides, or complexes on oxide supports. In the case of zeolite support, the active transition metal can be incorporated by ion exchange and subsequent processing to produce materials with appropriate porous, chemical and electronic properties. Such catalysts have sufficient long-term stability due, for example, to ionic linkages between the active centers and the crystal lattice (Bell, 2001).

Biomass is one of the important sources of energy after petroleum, coal and natural gas. It can be used as a renewable energy source and thus at the same time contribute to reduce organic wastes, tires, and plastic residues. Also, in the long term, biomass processes can be used for CO_2_ conversion/sequestration helping to reduce the temperature increase that causes climate change. An advanced design of catalysts, including TM/Z, can be relevant to develop new environmental benign biomass transformation processes. Also, the synthesis of biofuels or biochemicals is a promising area of research where TM/Z may play an important role to facilitate the next generation of ecological products. The new bioproducts obtained from residues and wastes can provide economic and social security while not competing with food resources such as sugars, starch or vegetable oils. The new generation of biofuels obtained from ligno-cellulosic rests, organic litters or forestry products do not impact food fabrication. For this purpose, improved catalysts that contribute to the selective production of desired biofuels and biochemical are necessary. Advanced research of such catalysts is in progress (Wang L. et al., 2017; Ren et al., 2019; Gopinath et al., 2020; Saab et al., 2020; Xia et al., 2021).

Not so long ago, a review was published devoted to the synthesis of catalysts based on transition metals supported on zeolites (Kosinov et al., 2018). In the present review, we focused on the latest process developments using similar catalysts to address current challenges in a variety of environmentally important and rapidly evolving processes in the fields of green chemistry, environmental protection and sustainable energy production. We also consider cases where the collaboration of theoretical and experimental research has helped to uncover the nature of specific catalytic processes. In addition, a Table 1 compiling several TM/Z-based catalysts associated with different catalytic processes is included at the end of this review article.

**TABLE 1:** Catalytic applications of distinct transition metals on zeolites (TM/Z).

Process	Zeolite	Transition metal	References
CO_2_ capture	Zeolite-4A, FAU, CHA, MER, RHO	Pd, Fe, Co, Ni, Cu, Zn, Ag	Sun et al. (2019), Debost et al. (2020), Panda et al. (2020), Kian et al. (2021)
CO_2_ conversion	MFI, BEA, CHA, LTA, USY	Fe, Co, Ni, Cu, Ru, Rh, Pt (promoters like Ce, Mg, Ca, Ba)	Sineva et al. (2015), Bacariza et al. (2017), Bacariza et al. (2018), Bacariza et al. (2019), Sadek et al. (2019), Ashok et al. (2020), Wei et al. (2021)
Conversion of methane to methanol	ZSM-5, ERI, CHA, MOR	Fe, Co, Ni, Cu, Rh, Au, Pd	Narsimhan et al. (2016), Paolucci et al. (2016), Bunting et al. (2020), Zhu et al. (2020b), Martini et al. (2020), Sajith et al. (2020), Pappas et al. (2021)
SCR-deNO_x_	MOR, BEA, MFI, FER, CHA, LTA	Fe, Co, Ni, Cu, Ag	Xin et al. (2018), Hamoud et al. (2019), Lee et al. (2019), Shelyapina et al. (2020b), Gao (2020), Gramigni et al. (2020), Lim et al. (2020), Lin et al. (2020), Ben Younes et al. (2021), Zhao et al. (2021)
H_2_ storage	ZSM-5, SSZ-13	Cu, Ag, Zn	Kozyra and Piskorz (2016), Ipek et al. (2018), Altiparmak et al. (2019)
H_2_ production	ZSM-5, Y-zeolite, USY, hierarchical-BEA, BEA	Ru, Pt, Rh, Pd, Co, Ni, Cu, Fe	Contreras et al. (2014), Li et al. (2019c), Luconi et al. (2019), Gogoi et al. (2020), Grzybek et al. (2020), Ogo and Sekine (2020), Wang et al. (2020)
Hydrocracking of plastic waste	H-ZSM-5, Y, FER, ITQ-6, ZSM-5-30, BEA-25, Y-30, REY, SAPO-11	Pt, Ni (Ce, La, and Mn as a promoters of Ni), Ga, Fe, Ce, Cu, Sn, Zn	Escola et al. (2012), Anuar Sharuddin et al. (2016), Zheng et al. (2017), Munir et al. (2018), Yao et al. (2018), Miskolczi et al. (2019), Al-asadi et al. (2020)
Pyrolysis of biomass	ZSM-5, H-ZSM-5, Y, β, SAPO-34, MCM-22, ITQ-2	Ga, Ni, Co, Sn, Rh, Zr	Cheng et al. (2012), Iliopoulou et al. (2012), Zheng et al. (2017), Naqvi and Naqvi (2018), Li et al. (2019a), Cai et al. (2020), Hernández-Giménez et al. (2021)
Transformation of natural oils and fats	meso-Y, SAPO-1, β, Hβ, micro-meso-β, ZSM-5, HZSM-5, MOR, A, BEA	Ni, Mo, Co, Fe, Cr, Sn, Zn	Dedov et al. (2016), Ji et al. (2017), Crawford and Carreon (2018), Ojeda et al. (2018), Cheng et al. (2019a), Asiedu and Kumar (2019), Cheng et al. (2019b), Zhang et al. (2019b), Crawford et al. (2020), Niu et al. (2020), Papanikolaou et al. (2020)
Oxy-dehydration of glycerol	ZSM-5, FAU, FER, MEL, MFI, MOR, MWW, BEA, BEA-Y, OFF, MCM-22	W, V, Mo, Fe, Fe modified by Rh	Diallo et al. (2016), Sánchez et al. (2016), Silva et al. (2017), Suganuma et al. (2018), Viswanadham et al. (2018), dos Santos et al. (2019), Ren et al. (2019), Possato et al. (2020)
Glycerol hydrogenolysis	Y, SAPO-11	Pt, Cu, Ir, Ru, Ni, Cu, Co, Zn	Gallegos-Suarez et al. (2015), Priya et al. (2016a), Priya et al. (2016b), Mitta et al. (2018), Li et al. (2019b), da Silva Ruy et al. (2020), de Andrade et al. (2020)
Aromatization of glycerol	HZSM-5, H[Sn, Al]ZSM-5	Mo, Zn, Ag, Ni, Cu, Sn, Fe, Nb, La	Wang et al. (2017a), Yang et al. (2018), Almeida et al. (2019), Lima and Perez-Lopez (2019)
Synthesis of bio-fuel additives from glycerol	ITQ-6, Y, USY, β, MFI, MOR	Au, Sn, Cu, Zn, Ag, Fe, W	Srinivas et al. (2014), Lari et al. (2015), Ozorio et al. (2015), Lu et al. (2017), Priya et al. (2017), Vieira et al. (2018), Zhou et al. (2019a)

## Catalytic Processes With Transition Metals on Zeolites

### CO_2_ Capture and Conversion

The use of fossil fuels in industrial processes and improper exhaust gas handling are the chief causes of pollution (Shelyapina et al., 2020a). Efforts are presently underway to mitigate CO_2_ emissions into the environment, with the aim of protecting the environment and public health. During the 1990–2014 period, global greenhouse gas emissions increased from 33.8 to 48.9 billion tons, including a 52% increase in CO_2_ emissions (Zhang J. et al., 2021). Although the greenhouse effect of CO_2_ is 25 times less than that of methane (Yin et al., 2010), the former significantly predominates over the latter. Therefore, CO_2_ is the major anthropogenically produced gas that contributes to global greenhouse gas.

CO_2_ can be separated and/or captured by different processes, including adsorption, membranes, cryogenics, and microbial or algae (Thiruvenkatachari et al., 2009; Elhenawy et al., 2020). In general, there are three capture categories: 1) after the combustion process (post-combustion); 2) previous to combustion (pre-combustion) and 3) the oxyfuel combustion capture (Wu et al., 2014). The affinity of zeolites for one of the components in a mixture of gases is due to the interaction of its acid sites and the quadrupole moment of the adsorbent molecule (CO_2_) (Graham et al., 1998). CO_2_ capture by highly porous materials can be regulated by adsorbate-adsorbent interactions (Jeong and Kim, 2016), and when this interaction is high, it may be considered as selective adsorption (Millward and Yaghi, 2005). The vast majority of research are devoted to the CO_2_ adsorption by materials such as metal-organic frameworks (MOF) (Millward and Yaghi, 2005; Zhang L. et al., 2021), covalent organic frameworks (COV) (Bagherian et al., 2021), based-carbons materials (Calvo-Munoz et al., 2016; Seema, et al., 2014), and zeolites (Jeong and Kim, 2016).

Zeolites possess excellent and tunable thermal stability and textural properties, rendering them promising for post-combustion CO_2_ capture. The creation of mesoporosity in zeolite 4A and its effect on the diffusion rate of CO_2_ was examined. Results showed that mesoporosity leads to reasonable CO_2_ adsorption capacity and to a decrease in moisture uptake (Panda et al., 2020). Faujasite (zeolite X) behaves similarly. The inclusion of palladium in 13X zeolite, causes absorption to rise up to 262.5 mg CO_2_ per gram of zeolite. Then, if this system is used as an absorbent for CO_2_ from steam methane reforming, this turns zeolites into potential materials to produce high purity H_2_ with low CO_2_ footprint (Kian et al., 2021). Similarly, much attention has been paid to small pore zeolites since they are attractive candidates due to the shape and size of the porous structures (Dusselier and Davis, 2018). High selectivity to CO_2_ over CH_4_ in small-pore zeolites such as CHA, MER, and RHO was observed (Debost et al., 2020). It was also found that the CO_2_ quadrupole moment has an impact, especially on cation-rich zeolites with a low Si/Al ratio, such as X zeolite, resulting in high capacity of CO_2_ capture (Sun et al., 2019).

The valorization of CO_2_ is a challenging topic due to its low reactivity and high stability. Not only removing CO_2_ emissions, but also converting them to other chemicals with higher added value requires high energy processes (Garba et al., 2021). CO_2_ can be converted to hydrocarbons. Valorization of CO_2_ by conversion to chemicals and hydrocarbons is an evolving path. At present, two approaches to CO_2_ conversion can be used; either CO_2_ can be reduced to a more reactive CO by a reverse water gas shift reaction (RWGS) followed by a Fischer-Tropsch reaction (FT) to form chemicals larger than C_1_ compounds, or the CO_2_ hydrogenation process.

The production of longer chains of C_2_ compounds can be carried out by hydrogenation of CO_2_. This process is also known as CO_2_-FT, since CO_2_ is hydrogenated to CO by RWGS, and then converted to hydrogenated compounds by FT. The FT reaction proceeds on heterogeneous catalysts containing Group VIII metals such as Fe, Co, Ni or Ru (Sineva et al., 2015). Cobalt and iron are the most explored metals of FT. However, iron is preferred because it is cheaper than cobalt, although Co possesses high selectivity to paraffins, low activity in water-gas shift, and it is less susceptible to sintering or deactivation by coke (Sadek et al., 2019). The most commonly used supports are SiO_2_, Al_2_O_3_, TiO_2_, ZrO_2_, zeolites, and MOF (Yang et al., 2017). A wide variety of zeolites such as ZSM-5, BEA, CHA (Fujiwara et al., 2015; Dang et al., 2018; Wei et al., 2021) have been used to support metals in this reaction.

**FIGURE 1:**
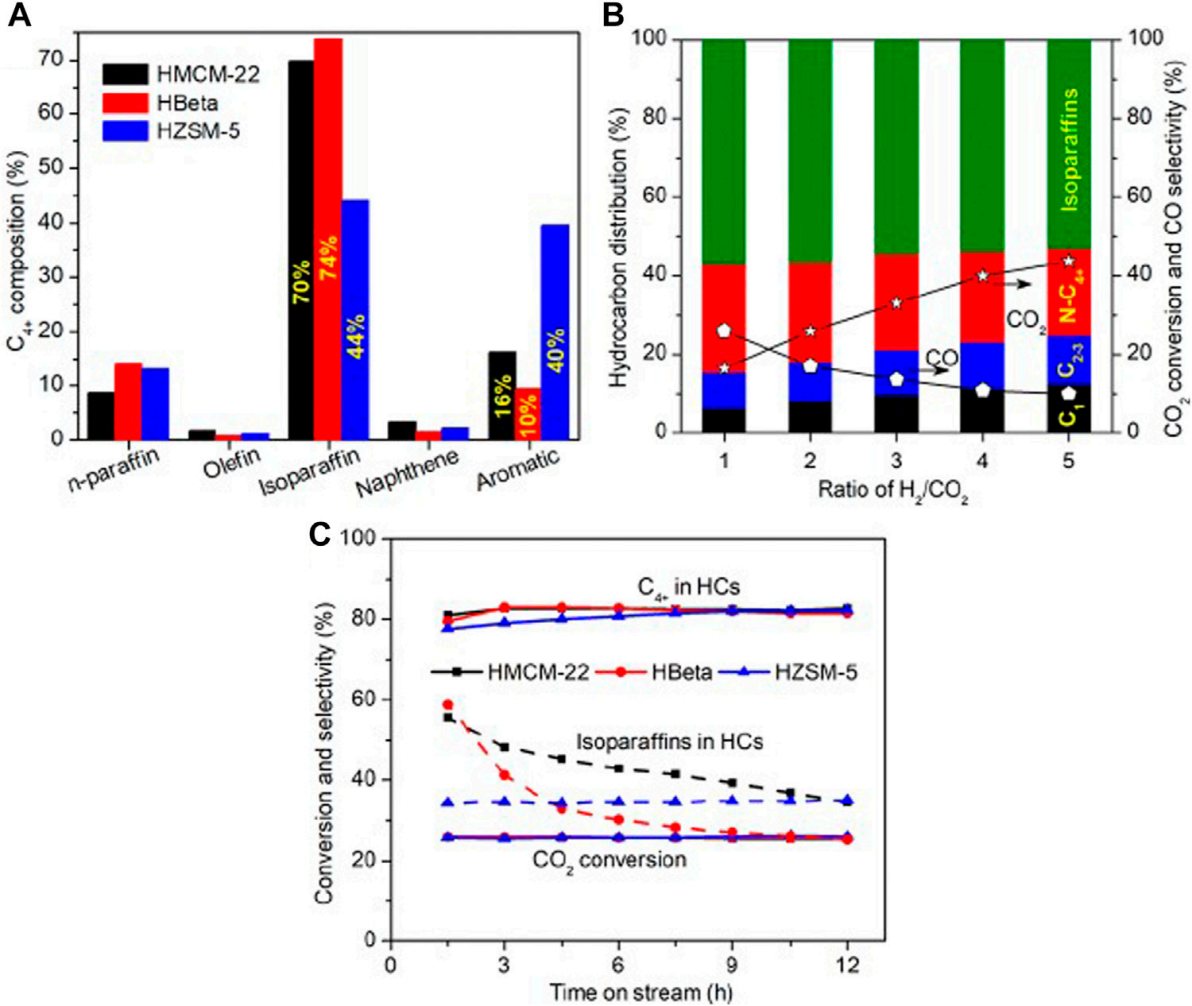
Catalytic performance of composite catalysts. **(A)** Distribution of products. **(B)** Conversion and selectivity. **(C)** CO_2_ conversion, C_4+_ and isoparaffin selectivity as a function of TOS. Reprinted with permission from Wei et al. (2018). Copyright 2018 American Chemical Society.

Metals have peculiarities in functioning for this reaction, and different selectivity for products; it is clear that this is highly dependent on the support used, and on the promoters of the catalytic system. In this reaction, iron proved to be a good catalyst for RWGS and FT, very selective for long-chain hydrocarbons, but if it is not promoted (for example, by potassium) it still presents selectivity for CH_4_ (Wang et al., 2011). Likewise, cobalt presents higher selectivity for heavier hydrocarbons than those iron-based catalysts (Dorner et al., 2010). It has also been reported that a bi-functional catalyst is needed, that is, a catalyst combining RWGS with FT activity to achieve the growth of an organic chain; TM with an acidic support can perform this task (Kang et al., 2008). In this regard, zeolites are especially relevant since the isomerization of hydrocarbons requires the presence of metal and acid sites in the catalyst (Přech et al., 2020). It has been suggested that the formation of mesoporosity in the MFI zeolite may improve the selectivity towards branched hydrocarbons, probably as a consequence of lowering the diffusional constraints. In this catalyst, cobalt nanoparticles exhibit resistance to sintering, high CO conversion, and a long catalytic lifetime (Kim et al., 2014). Recent reports suggest that the particular steric and confinement properties of zeolites can be responsible for the selectivity for C_5_ (and larger) products and iso-paraffins (Wei et al., 2018) (Figure 1), which can be produced by oligomerization of shorter chains (Noreen et al., 2020). The well-known features of zeolites in organic synthesis, such as acidity and shape selectivity, are also very important for synthesizing aromatic compounds. Some studies have reported that the moderate distribution and stronger Brønsted acid sites (BAS) are good for synthesizing light aromatics, while a high density of BAS can cause major coke formation (Wei et al., 2021).

A theoretical study has shown that using a membrane reactor instead of a traditional one, the CO_2_ to methanol reaction would be favored over the RWGS, when a selective separation of methanol is taken into account by a separation factor. Besides, the performance of the system is improved with a hydrophilic zeolite membrane (Barbieri et al., 2002). An experimental study found that an LTA zeolite membrane reactor performs better than a conventional reactor, showing good conversion and selectivity to methanol (Gallucci et al., 2004).

The second process, which consists in direct conversion of CO_2_ to chemicals by hydrogenation, can be achieved similarly to FT, but instead of CO, it uses CO_2_ and therefore, more hydrogen is needed. The CO_2_ hydrogenation process can produce various products such as methane, methanol, gasoline (C_5_–C_11_), and iso-paraffins, among others. The conversion of CO_2_ to methane is known as methanation. The more common metals tested in methanation are Ni, Rh, Ru, Pt, and some promoters like Ce and Mg, are supported on various supports like Al_2_O_3_, TiO_2_, CeO_2_, ZrO_2_, SiO_2_, mesoporous SiO_2_, among others, as published in recent reviews (Bacariza et al., 2019; Ashok et al., 2020).

The influence of the most important characteristics of zeolites, such as the Si/Al ratio, the type of the extra-framework cation, and the hydrophobicity/hydrophilicity characteristics, were evaluated (Bacariza et al., 2017; Bacariza et al., 2018). A higher Si/Al ratio was found to favor greater catalytic activity. The higher performance in the methanation process could be a consequence of the higher capture of CO_2_ observed in zeolites with a low Si/Al ratio, meanwhile, the high hydrophobicity of the zeolite has a detrimental effect on the methanation. Bacariza et al. (2018) showed that the presence of water molecules can inhibit CO_2_ methanation when the Si/Al ratio decreases. Likewise, previous works reported about the effects of monovalent and divalent exchangeable cations present simultaneously with TM cations (Bacariza et al., 2017). For Ni supported on USY, the addition of various monovalent cations, gave the following trend Cs^+^ > Na^+^ > Li^+^ > H^+^ for methanation, while for divalent cations such as Ca^2+^, Ba^2+^ and Mg^2+^; the latter was the best for improving the catalytic performance (Bacariza et al., 2017). Copper catalysts are being actively researched for the production of methanol from CO_2_ due to the similarity of the reaction to methanol production from syngas.

### Conversion of Methane to Methanol

The growing production of natural gas and the so-called “shale gas revolution” has sparked discussions about its advantages and disadvantages, as well as about emerging technical problems important for its rational use (Boersma and Johnson, 2012; Grecu et al., 2018; Wang, 2018; Wang et al., 2019; Gao et al., 2021). Raw natural gas contains fractions such as CH_4_, H_2_S, CO_2_, C_2_H_6_, and other light alkanes, which are valuable either as chemical feedstock or as fuels (Shah et al., 2016). Since methane is the main component of natural gas (70–90%) (Lunsford, 2000; Faramawy et al., 2016; Schwach et al., 2017) and it is 25 times more efficient in greenhouse effect than CO_2_ (Yin et al., 2010), it is obvious that a process needs to be found to convert it into high value-added products.

Methanol is an important feedstock for many industrial processes, but it can also be used as fuel or to increase the octane rating of gasoline. Also, methanol can be converted to gasoline and chemicals by some catalytic processes. The most explored method is conversion to synthesis gas (“syngas”) (indirect route), which is based on a two-stage process: 1) conversion of methane to syngas and 2) synthesis of hydrocarbons using the FT reaction, or methanol synthesis followed by conversion to oxygenated hydrocarbons (Loricera et al., 2017). The direct route is a single-step process wherein methane interacts with oxygen or other oxidizing agents to produce the desired product, methanol or formaldehyde. Energetically, it is the most efficient, and therefore the most desirable. The conversion of methane to methanol (MTM) by a partial oxidation reaction stands as a holy grail of catalysis (Arndtsen et al., 1995; Zhou Y. et al., 2019). On the other hand, partial oxidation of methane can lead to both methanol and carbon monoxide, which are exothermic processes. However, these processes occur at temperatures above 600 K, where CO oxidation is greatly accelerated (Loricera et al., 2017). Therefore, it is imperative to find catalytic materials for the activation of methane at temperatures below 500 K (Li et al., 2016; Sushkevich et al., 2017).

The use of molecular oxygen as an oxidizing agent is crucial to the competitiveness of any direct MTM conversion technology, since molecular oxygen is cheap and available (Edwards and Foster, 1986). Indeed, several compounds playing the oxidant role have been tested, namely, N_2_O (Dubkov et al., 2002; Ipek and Lobo, 2016), H_2_O_2_ (Hammond et al., 2013; Hutchings, 2016), O_2_ (Wulfers et al., 2015; Shan et al., 2017; Bozbag et al., 2018), and H_2_O (Sushkevich et al., 2017). Methane and methanol molecules have singlet fundamental states, and their oxidation by O_2_ is a spin-forbidden reaction. Therefore, an appropriate choice of catalyst is required to activate the reaction (Schwarz, 2011). In this sense, the option of a solid catalyst seems to be an attractive route. However, methanol has a higher dipole moment than methane; consequently, it is preferentially activated on the surface of a catalyst, promoting its over-oxidation. The preferential methanol adsorption on the catalyst surface implies an additional step to recover the methanol by extraction by a polar solvent. In this sense, one of the biggest challenges in the conversion of methane to methanol is finding a suitable catalyst that will be able to adsorb and only partially oxidize CH_4_ and then facilitate the desorption of the products. Due to the complexity of this last step, extensive research continues to be promoted in the field.

The catalysts based on zeolites exchanged with transition metals have attracted significant attention as they present high selectivity to methanol. Microporous zeolite materials are characterized by the fact that they crystallize in a wide variety of structures, have a remarkable ability to exchange cations, and have the capacity to support active sites with controlled nuclearity. Although different metal cations such as Co, Ni, Rh, Au, Pd, etc., can be housed in zeolites (Shan et al., 2014; Krisnandi et al., 2015; Bunting et al., 2020; Sajith et al., 2020), research has shown that the most promising results correspond to the inclusion of iron or copper. The first efforts were made by Pannov et al. in the early 90s’ (Pannov et al., 1990; Panov et al., 1993). The iron species in the cavities of ZSM-5 zeolite can coexist as mononuclear, binuclear, oligonuclear cationic species, or as neutral iron oxide species (Sun et al., 2008). However, the active site in the methane to methanol reaction was determined to be a mononuclear Fe(II) species formed by irreversible auto-reduction of impregnated Fe(III) species after heat treatment (Starokon et al., 2011). Another outstanding work by Groothaert et al. (2005), showed that Cu-exchanged zeolite ZSM-5 can catalyze the methane oxidation reaction in the Cu-ZSM-5 system after activation in O_2_ or N_2_O. Copper exchanged zeolites have shown similar reactivity to methane oxidation compared to Fe exchanged zeolites, but their advantage is that O_2_ can activate them. In this sense, copper exchanged in different zeolite structures has emerged as one of the most exciting systems. Other zeolite structures such as ERI, CHA, MFI exchanged with copper have also been evaluated in the MTM reaction (Beznis et al., 2010; Paolucci et al., 2016; Martini et al., 2020; Zhu et al., 2020b); however, the Cu-MOR system remains one of the most promising. Notwithstanding, the most suitable site for partial oxidation in the MTM reaction continues to be a matter of discussion (Groothaert et al., 2005; Mentzen and Bergeret, 2007; Woertink et al., 2009; Smeets et al., 2010; Grundner et al., 2015, 2016; Narsimhan et al., 2016; Pappas et al., 2021).

**FIGURE 2:**
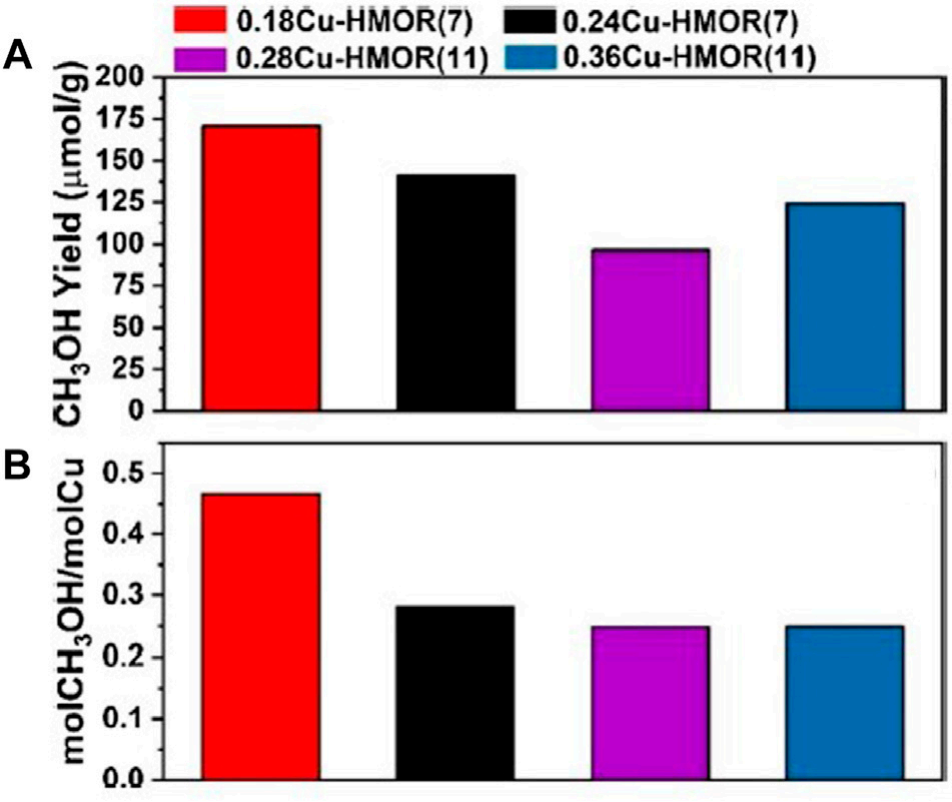
**(A)** CH_3_OH yield in μmol/g and **(B)** normalized productivity in mol_CH3OH_/mol_Cu_. Reprinted with permission from Pappas et al. (2021). Copyright 2021 American Chemical Society.

Despite the promise of catalytic systems based on zeolites exchanged with iron and/or copper, their performance remains low. The methanol productivity normalized to Cu content is one of the typical values reported in the literature for both Cu-ZSM-5 and Cu-MOR systems, whose dimensions correspond to those of mole of methanol per mole of copper (mol_CH3OH_/mol_Cu_). Some of the highest reported values are 0.20 (Narsimhan et al., 2016) and 0.47 (Pappas et al., 2021) mol_CH3OH_/mol_Cu_ for the Cu-ZSM-5 and Cu-MOR systems. This would suggest that if we follow the idea that only one copper atom is needed to produce one molecule of CH_3_OH, only one part or one tiny fraction of the copper atoms participate in the partial oxidation of methane. On the other hand, we can assume that the yield of the catalytic system is a sign of how many atoms are forming the active site. In this sense, Grundner et al*.* suggest that in the case of the Cu-MOR system the 0.35 mol_CH3OH_/mol_Cu_ yield is a clear proof of the presence of a trinuclear copper active site (Grundner et al., 2015; Grundner et al., 2016). In addition, Pappas et al. (2021) reported a value close to 0.5 mol_CH3OH_/mol_Cu_ (Figure 2), suggesting that the nature of the active site is binuclear. Thus, it is evident that further and more detailed investigation is required to clarify the phenomenology in depth.

### Selective Catalytic Reduction of NO_x_ (SCR-deNO_x_)

Since Iwamoto et al. (1986), Iwamoto and Yahiro (1994) showed more than 20 years ago that Cu supported on ZSM-5 proved to be a highly active catalyst for the reduction of nitrogen oxides, selective catalytic reduction (SCR-deNO_x_) by transition metals supported on zeolites has become a widely studied technology for vehicles powered by internal combustion engines, and stationary sources (Wang J. et al., 2017; Xin et al., 2018; Han et al., 2019). The most studied transition metals are groups IB and VIIIB, mainly copper, iron and cobalt (Sazama et al., 2016; Saeidi and Hamidzadeh, 2017; Zhao et al., 2021). The main zeolite frameworks used as supports for these catalysts are MOR, BEA, MFI, FER, and CHA, because of their thermal stability, high surface area, availability of exchange sites, the potential for clusters stabilization, among other intrinsic properties that make these materials excellent supports (Ben Younes et al., 2021). However, the synthesis, design, and optimization of the catalysts to achieve the high activity, selectivity, and stability remain a challenge. Although the trend towards the use of clean energy and zero carbon emissions will clearly increase in the coming decades, diesel engines will continue to be the primary means of transporting heavy loads over long distances; hence the need for the development of more efficient technologies to mitigate emissions of nitrogen oxides from these sources will continue to be a topic of interest. This chapter will focus on SCR-deNO_x_ with hydrocarbons or ammonia for diesel engines with transition metals supported on zeolites.

Likewise, several parameters have been studied during the synthesis of the catalysts. The most recently studied are: metal deposition method, metal loading, and order of metal deposition for multi-metal materials. Regarding the methods of deposition of TM/Z catalysts, 1) the liquid-state ion exchange or conventional, 2) the solid-state ion exchange, and 3) the incipient impregnation stand out from other ones. In general, the preparation method affects the loading of the metal; among the methods, ion exchange with aqueous solutions prevails. Recently, Lin et al. (2020) analysed the effect of the preparation method on the catalytic activity of Cu/LTA on SCR-deNO_x_ with NH_3_. They found that the conventional ion exchange carried out with a 0.01 M copper acetate precursor solution presented the highest catalytic activity in NH_3_-SCR above 400°C, which was associated with the presence of Cu(II) ion species. Likewise, Lee et al. (2019) studied the effect of Cu loading supported on SSZ-13, ZSM-5 and BEA zeolites in the SCR-deNO_x_ with C_3_H_6_. The CuZSM-5 catalyst with 2 wt% of metal presented the highest NO_x_ conversion (68% at 360°C) compared with Cu/SSZ-13 and Cu/BEA catalysts with different Cu loadings. Authors concluded that the NO_x_ reduction was influenced by metal loading and topology of the zeolite, the active sites being mainly associated with the presence of isolated Cu^2+^ ions. Zhang L. et al. (2019) reported new methods of preparation of zeolites for application in SCR-deNO_x_, and they concluded that environmentally friendly synthesis methods can be convenient to prepare these supports. Lately, it was reported that the order of deposition of metals during the synthesis of the TM/Z catalysts, clearly influences the catalytic performance. Such effect is associated with redox interactions between metal species that lead to different types of active phases according to the order of deposition (Jouini et al., 2018; Aziz et al., 2020; Shelyapina et al., 2020b; Sánchez-López et al., 2020).

Others parameters related to achieve optimal operation conditions of the SCR-deNO_x_ process with TM/Z catalysts include: the type of reducing agent (light hydrocarbons or ammonia) (Heo et al., 2019), and the type of atmosphere (oxidizing or reducing) (Gu et al., 2015). For instance, Lim et al. (2020) studied the SCR-deNO_x_ with CH_4_ under wet conditions with cobalt supported over different framework zeolites (small-pore). Results showed that catalytic activity of Co-CHA, Co-RTH, Co-UFI and Co-LTA with similar Co/Al ratio, decreased in the presence of water. Within these zeolites, chabazite was the most resistant to water vapour during CH_4_-SCR. In any case, it was concluded that both the topology of the framework and the Si/Al ratio are determinants for the catalytic performance.

According to Mytareva et al. (2021) one of the new trends in the development of catalysts to reduce NO_x_ is to increase efficiency at low temperatures. As has been well demonstrated, the highest catalytic performance of TM/Z catalysts is achieved in the temperature range of 300–450°C, with NO_x_ conversion higher than 60% and N_2_ selectivity around 70%. However, in most cases the efficiency at low temperatures (<300 °C) is even lower, so an important objective in optimizing of these catalysts will be to break this limitation. Recently, Lomachenko et al. (2016) studied a typical catalyst for SCR-deNO_x_ based on Cu-CHA, which was monitored by operando XAS and XES as a function of the reaction temperature (150–400°C). They found that the catalytic activity at high and low reaction temperatures was due to different Cu species. The activity at low temperature was related to a mixture of Cu(I)/Cu(II) sites. Then, in order to improve the catalytic activity over a wide temperature range is necessary to establish the optimal proportion of active species, in mono and multi-metallic catalysts. In several review works, for low-temperature SCR-deNO_x_ catalysts (Guan et al., 2014; Stakheev et al., 2015; Gao, 2020; Gramigni et al., 2020), the authors concluded that zeolites with small and large pores such as; BEA, MFI, CHA, LTA, etc., with Fe and Cu mainly, have shown the best performance in the operating range less than 300°C.

For instance, mono- and multimetallic catalysts have shown dynamic behaviour and different active sites during the SCR-deNO_x_ due to the appearance of different metal species in the zeolite frameworks such as isolated ions (monomeric), dimers, multimeric species, and clusters in various oxidation states, metal nanoparticles and more recently the so-called single-atoms that may act as active centers. Therefore, these materials are considered “dynamic catalysts” because they carry out the NO reduction reaction by redox cycles changing the oxidation state. It has been discussed by Liu and Corma (2018), various sizes of metallic species provide different behaviour during the catalytic evaluation, mainly due to their electronic differences and dynamic redox characteristics. Recent studies on stabilized sub-nanometric metal clusters in the cavities of zeolites have shown good catalytic performance compared with the properties of bigger metal nanoparticles.

Cu and Fe supported on various zeolites have been widely studied to raise, the different active sites and their potential for improving the efficiency in SCR-deNO_x_. *In-situ*spectroscopic studies of Cu and Fe for SCR-deNO_x_ revealed in detail the different species active during the reaction (Skarlis et al., 2014; Borfecchia et al., 2018; Negri et al., 2018; Fahami et al., 2019; Bergman et al., 2020; Zhang et al., 2020). Thus, Liu et al. (2020) studied Cu-CHA catalysts for NO_x_ reduction with *in-situ* spectroscopy and on-line mass-spectroscopy to analyze Cu(I)/Cu(II) redox cycles during the catalytic test. According to their results, the Cu(II) sites act at low temperatures (<200°C), while at high temperatures, the Cu(I)/Cu(II) sites act together. Moreover, studies with *in-situ* FTIR spectroscopy carried out by Hamoud et al. (2019) on Cu-Fe catalysts supported on CHA and MOR zeolites revealed that the Cu(I)/Cu(II) sites are most active at low temperatures, while the Fe(II)/Fe(III) species were more active above 300°C. It is clear that the discussion on the most active copper or iron site for the SCR-deNO_x_ is still ongoing. However, techniques such as *in-situ* spectroscopies and theoretical modelling can shed light on the reaction mechanisms of these catalysts.

Finally, the state-of-the-art research of catalysts based on transition metals supported on zeolites for SCR-deNO_x_ for a better generation of catalysts must imply the optimization of: 1) the synthesis parameters, 2) the optimal ratio of the active phases and 3) the reaction conditions emulating the sources emission. In this sense, tools such as machine learning or artificial intelligence will identify the optimal conditions for the development of catalytic materials in the near future. An example of this is the research carried out by Jensen et al. (2019) with the support of predictive models and data science to find the best route of synthesis of materials. In that work, the authors studied the parameters for the synthesis of zeolites with machine learning support to extract the best synthesis conditions. The extraction of parameters was obtained from a database of 70,000 articles of zeolites synthesis that were processed. They concluded that it is possible to get a new generation of materials by optimizing synthesis parameters by data mining and machine learning.

### Catalytic Upgradation of Biomass and Polymeric Waste

**FIGURE 3:**
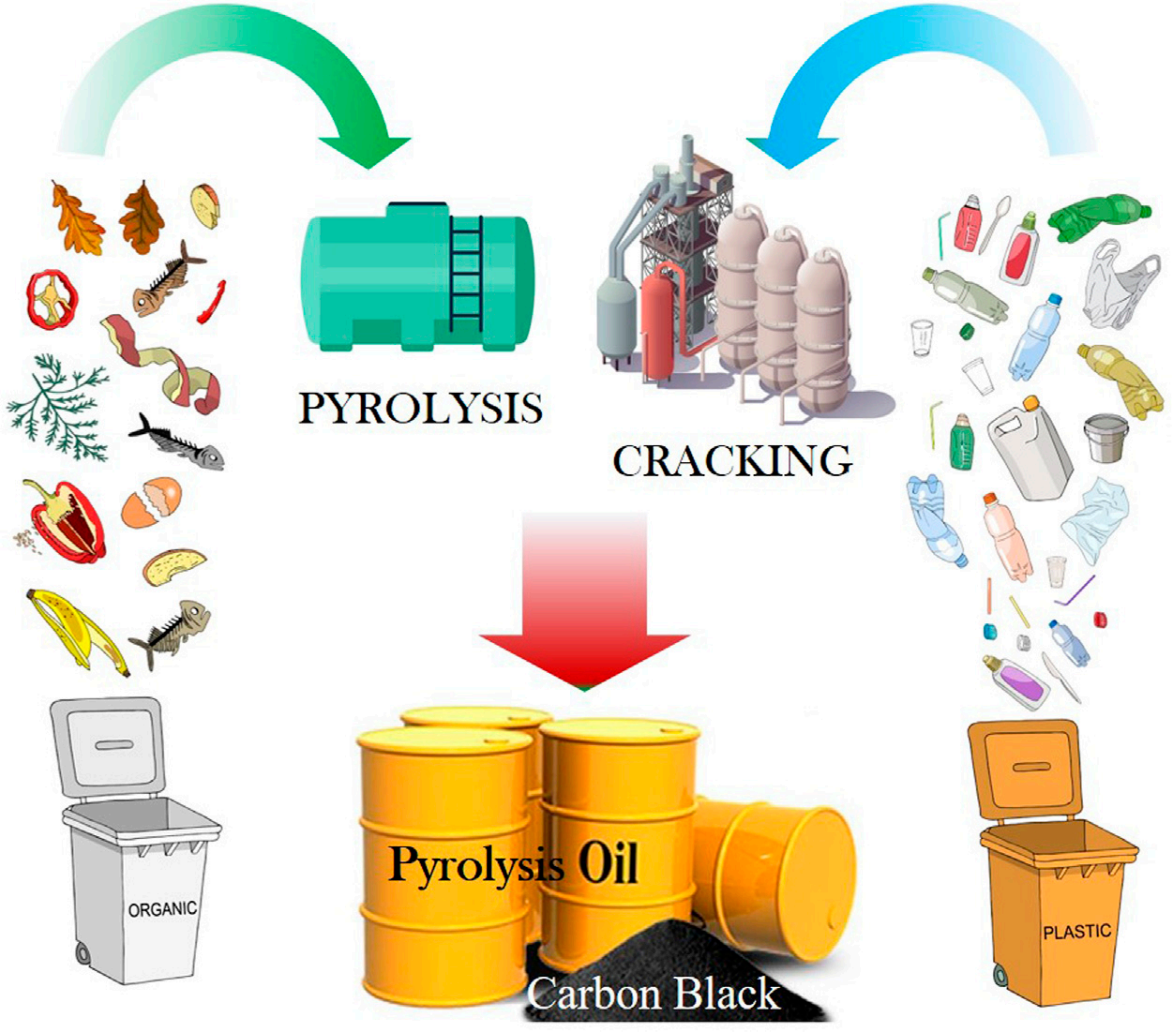
Catalytic degradation of polymeric and organic waste.

The generation of solid waste is an integral part of human life as a consequence of industrialization, and has a serious impact on the environment. Nowadays, due to growing demand and limited resources, utilization of waste is becoming increasingly important as a potential raw material for energy and chemical production (Figure 3). Plastic waste is widely available, and the cost of its disposal is partially offset by the cost of the products derived from it (Corma et al., 2007). Waste treatment technologies rely on recycling and degradation. Degradation of mixed or separated municipal solid waste (plastic, biomass, paper, rubber, textiles) is considered an important method from which fine chemicals and fuels can be produced. Degradation technology includes several stages and depends on the feedstock composition and is achieved with photo-, thermo-, mechanochemical or catalytic degradation (Wang L. et al., 2017). In the present chapter, various methods of catalytic waste degradation (CWD) will be considered.

Zeolites are used in typical polymer degradation processes such as cracking and isomerization due to their acidic nature, and a deposited TM plays a key role in pyrolysis and coking reactions. However, to apply them in CWD the following points need to be considered: 1) simultaneous handling of waste of varied composition; 2) efficient deoxygenation of the oxygen-containing organics; 3) high resistivity towards coking formation, and 4) generation of liquid products with a low boiling point, and a higher ratio of iso-alkane to *n*-alkane, and olefins (Zheng et al., 2017). The most striking point of TM/Z in CWD is their high propensity to form expensive cyclic and aromatic compounds. At the same time, easy deactivation due to the formation of coke is a big disadvantage of zeolite catalysts. However, zeolite deactivation during CWD could be overcome by metal loading.

It is known that the efficiency of CWD reactions relies on several intrinsic (catalyst-related) and extrinsic (process-related) factors. The intrinsic factors are as follows:

• The acidity, the types of acidic sites, and their balanced distribution are the major factors to be considered in catalyst design.
• The pore size distribution determines the accessibility of the reactants and reaction intermediates to the active sites.
• The morphology, structure, Si/Al ratio, and general chemical composition etc., have a significant impact on the catalytic degradation yield. However, their influence is not directly on the active centers but on other intrinsic factors (Wang L. et al., 2017).
• The second component deposition [like noble metals, non-noble metals of group VI-A or VIII-A of the periodic table and metal oxides/carbides/nitrides (Ogo et al., 2017; Munir et al., 2018; Saab et al., 2020)] on the surface of zeolites can significantly influence the degradation reaction.

Moreover, the stability of a zeolite depends on the transition metal ion, the topology of the zeolite framework, its composition (Si/Al ratio), the type and conditions of the reaction, and the specific solvents and substrates used in the reaction. Silanol defect sites are one of the main factors affecting structural stability during hydrothermal treatments (Zhang et al., 2015). Under hydrothermal conditions, the deactivation of the zeolite could be overcome by increasing the hydrophobicity of the zeolite by silylation (Corma et al., 2003), increasing the susceptibility of transition metal ions under the reaction conditions (Yang et al., 2013), using low polar solvents (Lewis et al., 2014). For example, M-BEA zeolite is one of the strong water inhibitors that can be used to transform biomass in several reactions (Luo et al., 2016).

It should be remembered that all of the mentioned intrinsic factors depend on the origin of feedstock—one of the extrinsic factors. In the present chapter, the advantages and disadvantages of the TM/Z catalysts in CWD of biomass and plastic feedstock were considered.

#### Pyrolysis of Biomass

Biomass consists mainly of three polymers: cellulose, hemicellulose, and lignin. Therefore, various chemicals can be produced (Corma et al., 2007) by depolymerization or pyrolysis of biomass. Catalytic fast pyrolysis (CFP) of renewable lignocellulosic biomass represents a simple, cheap, and efficient approach to produce bio-based fuels and chemicals. During thermopyrolysis solid biomass is subjected to high temperature (500–700°C) and converted into light gases (CO, CO_2_), solid char, and liquid pyrolysis oil. However, the presence of oxygenated compounds (aldehydes, ketones, furans, carboxylic acids, phenolic compounds etc.) leads to high viscosity and low stability, complex structure, and preventing the direct application of pyrolysis oil. To overcome this, CFP with TM/Z catalysts are identified as one of the most promising (Veses et al., 2015; Hoff et al., 2016).

In cracking and aromatization reactions, the acidity and ideal pore size of zeolites with enhanced accessibility to acid sites remarkably increases the aromatics formation. CFP using ZSM-5 zeolite as a catalyst produces BTX, and naphthalene, which are known as building blocks of the petrochemical industry. Several zeolites (Y, *β*, ZSM-5, SAPO-34, MCM-22, ITQ-2, etc.) were applied for CFP and the best performance was demonstrated by ZSM-5 with high yield of aromatics rather than aliphatic hydrocarbon and significant percentages of deoxygenation (Zheng et al., 2017; Naqvi and Naqvi, 2018; Li J. et al., 2019; Cai et al., 2020). CFP on ZSM-5 is currently the most studied and is already employed in refineries (Jae et al., 2011; Taarning et al., 2011; Kubička et al., 2013). However, the lack of structure-activity correlations currently constitutes a major barrier for the rational design of ZSM-5 catalysts for CFP (Hoff et al., 2016).

The presence of metal species always promotes deoxygenation, improves the pyrolysis oil yield with minimal coke formation, etc. Iliopoulou et al. (2012) reported that NiO and Co_3_O_4_ nanoparticles supported on ZSM-5 have a positive effect on the deoxygenation process with increased aromatics yield. Cheng et al. (2012) demonstrated that Ga, Ni, or Sn oxides deposited on H-ZSM-5 leads to high production of hydrocarbons while Ga_2_O_3_/H-ZSM-5 promotes decarboxylation and olefin aromatization pathways. The use of mesoporous ZSM-5 with Ru NPs significantly increased the yield of alkane selectivity. However, one limitation of the use of mesoporous zeolites is the decrease in acidity i.e., reducing the activity (Wang L. et al., 2017). ZrO_2_ supported on the nano-crystalline ZSM-5 demonstrated promising activity, while its stability left much to be desired due to reversible crystallinity distortion by coke formation and re-dispersion (Hernández-Giménez et al., 2021).

Thus, successful commercialization of CFP could be possible with TM/ZSM-5-based catalyst. However, several technical obstacles need to be considered, such as continuous reactor design, process optimization, and product selectivity, among other factors, that also need to be taken into account (Chang et al., 2018).

#### Hydrocracking of Plastic Waste

The conventional regulations of plastic waste disposal, such as landfill and recycling, are inadequate due to environmental and economic inefficiencies. Therefore, an alternative for this is the utilization of plastic waste by liquefaction, biological treatment, and pyrolysis (Al-Salem et al., 2010; Sharma et al., 2015; Lopez et al., 2017). Pyrolysis of plastic waste has been explored to convert plastic waste into fuel at high temperatures, while using a catalyst enhances the degradation and reduces the energy requirements (Yuan et al., 2014; Jin et al., 2019; Gopinath et al., 2020). In very recent studies, pyrolysis has also been performed in the presence of hydrogen to minimize the extent of coking and the fractional yield of unsaturated compounds (Bai et al., 2019). The most widely used catalysts for pyrolysis are H-ZSM-5 (Munir et al., 2018), Y-Zeolite (Anuar Sharuddin et al., 2016), and silica-alumina crystals held in a zeolite matrix, for the production of high yields of liquid products and catalysts are easily regenerated by steaming (Syamsiro et al., 2014).

For the hydrocracking reaction, a catalyst with a strong acidic function (zeolite), and strong hydrogenation-dehydrogenation function provided by the supported TM, is used. Furthermore, the porosity of the TM/Z plays a pivotal role in plastic waste hydrocracking. In the polymer cracking over the conventional microporous zeolites, the major products consist of gases and a minor quantity of liquids due to the diffusion limitation of larger molecules through the pores. This is overcome by the use of mesoporous zeolites that increase the yield of liquid molecules rather than gaseous molecules. However, mesoporous zeolites generally have weak hydrothermal stability and reduced surface acidity, leading to a drop in catalytic activity. The large polymer molecules need to be cracked initially and then reformed by hydrocracking to obtain an increased liquid product with reduced gaseous contents. Therefore, the mesoporous structures need to be improved in the zeolite with a desired acidity. Hence, micro- and mesoporous composite catalysts are desired for polymer processing (Olazar et al., 2009; Serrano et al., 2012). Therefore, based on the feedstock type and yield requirements, bi-functional zeolite-based catalysts needs to be well designed to overcome the limitations of conventional zeolites. Bi-functional Pt supported on FER (microporous) and ITQ-6 (delaminated) catalyst was successfully applied for poly-styrene hydrocracking, yielding low aromatics (Munir et al., 2018).

The oil obtained from the thermal cracking of low-density polyethylene (LDPE) mainly consists of linear hydrocarbons with a high quantity of olefins, which hinders the possible application of this product in the formulation of transportation fuels. However, hydro-reforming of this oil using TM/Z catalysts would allow for the production of gasoline and diesel fractions. Conversion of polyethylene into transportation fuels by combining thermal cracking and catalytic hydroreforming over hierarchical BEA zeolite (with a bimodal micro-mesoporosity) with 7 wt% Ni has proved to be an efficient catalyst for obtaining gasoline (Ding et al., 1997). Catalytic steam reforming of waste high-density polyethylene for the production of hydrogen/syngas has been investigated using different zeolites (ZSM-5-30, BEA-25 and Y-30) supported nickel catalysts in a two-stage pyrolysis-catalytic steam reforming reactor system, where the Ni/ZSM-5-30 catalyst generated the maximum syngas production with excellent coke resistance and thermal stability (Escola et al., 2012).

For mixed plastic waste (MPW) pyrolysis, zeolite-based nickel catalysts are successfully applied since Ni has good catalytic properties for cleavage of C-C, O-H, and C-H bonds of oxygenated organics. The most effective catalyst of combined pyrolysis reforming is rare-earth metal exchanged Y-type (REY) zeolite ion-exchanged with nickel due to the proper pore size and acidic properties. The optimum amount of nickel loading on REY catalyst was 0.5 wt% due to the high yield of quality gasoline (Yao et al., 2018). Ni oxide supported on ZSM-5 or SAPO-11 reduces the content of large chains, accelerating the rate of the cracking reaction during MPW pyrolysis, and increasing the amount of volatiles with a minimum coke content. The increase of the Ni content reduced the yield of wax which in turn increased pyrolysis oil yield (Zheng et al., 2017). In another report, Ce, La, and Mn were used to promote the Ni/ZSM-5 in syngas production from MPW (polyethylene, polypropylene and terephthalate polyethylene). The modified catalysts enhanced the pyrolysis reaction rate resulting in high syngas yields. These catalysts can also accelerate methanization reactions and isomerize the main carbon chain. Furthermore, using Ce and La-promoted catalysts increased temperature and oxygen in the atmosphere and had a positive effect on syngas yield (Songip et al., 1995).

The selectivity of the aromatics during co-pyrolysis of lignin and polyethylene can be controlled by adding different metals on the surface of the zeolite. The presence of Ga increased the selectivity of mono-aromatics and at the same time converted the propane into olefins by the pyrolytic cracking route. However, Ga-containing zeolite with a smaller pore size leads to catalyst deactivation due to formation of large polyaromatics, and as a result, the catalyst is deactivated (Zheng et al., 2017). For chlorine-contaminated MPW sources, the H-ZSM-5 with gallium oxides sustained catalytic activity for a longer period than parent zeolite (Al-asadi et al., 2020). Another known modifier for chlorine-contaminated MPW sources is Fe. Due to its capacity to react with Cl and Br leading to a decrease of halogen content in MPW pyrolysis products (Zheng et al., 2017). ZSM-5 and Y-zeolite catalysts loaded with polyvalent metal ions (Ce^2+^, Cu^2+^, Fe^2+^, Fe^3+^, Ni^2+^, Sn^2+^, and Zn^2+^) were tested in a waste end-of-life vehicle pyrolysis. Irrespective of the zeolite type (ZSM-5 or Y), the TM/Z catalysts decreased the activation energies for decomposition and the activation energy decreasing order was as follows: Cu < Ce < Ni < Fe(III) < Fe(II) < Zn < Sn, however, the effect is more pronounced in the case of Y-zeolite based catalysts than ZSM-5 (Miskolczi et al., 2019).

The literature reveals that; the original zeolites are much more often used for the processing of plastic waste. A narrow range of TM has been investigated as modifiers of zeolite-based catalysts. However, the broad prospects for the development of TM/Z catalysts of plastic waste pyrolysis have recently attracted increasing attention of researchers.

#### Transformation of Natural Oils and Fats

**FIGURE 4:**
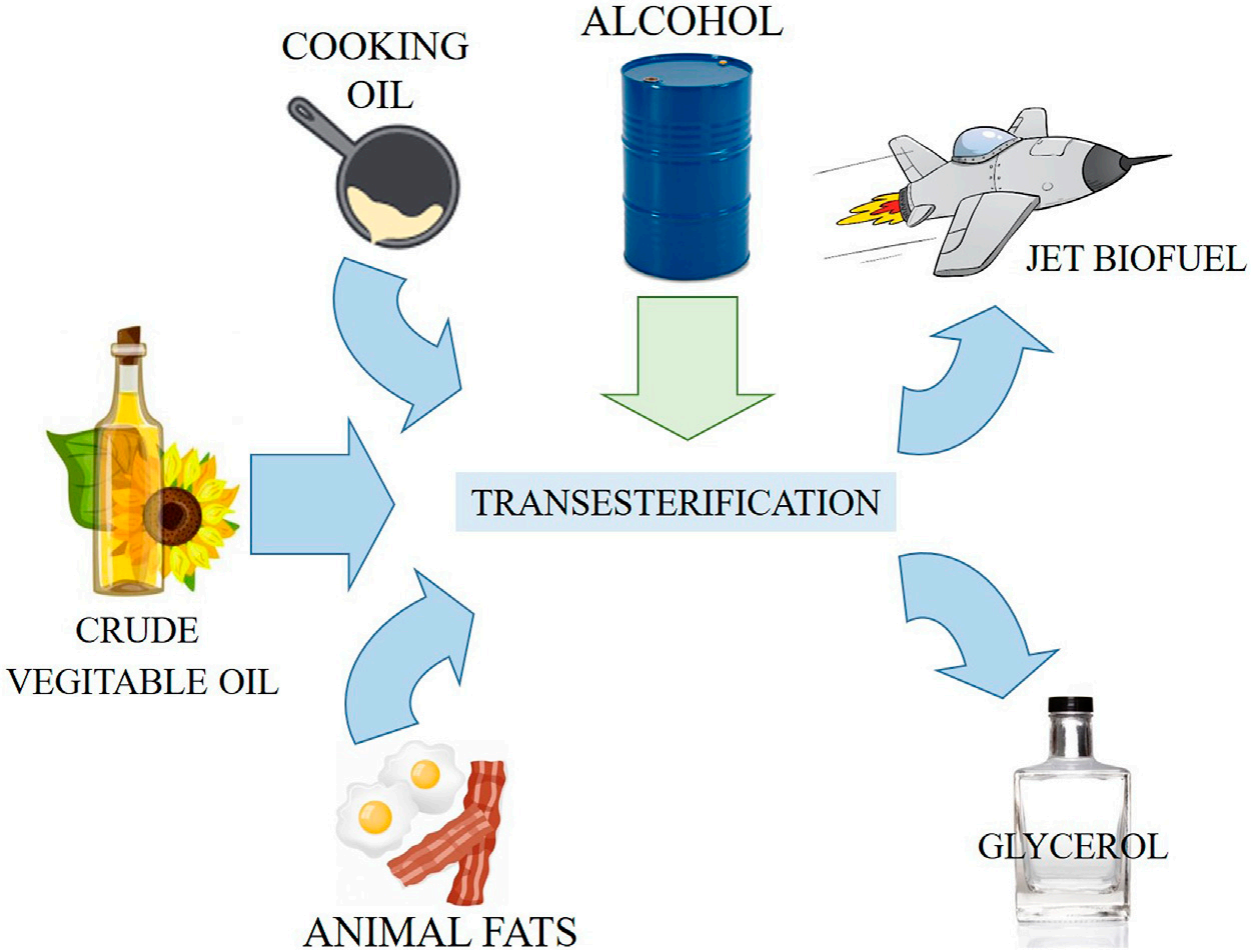
Catalytic transformation of natural oils and fats.

Natural oils and fats (NOF) have several reactive groups that can be converted into desired products. Oils in particular are considered suitable sources for jet fuel production due to their low content of aromatic substances. A multi-stage strategy for converting lipids into reactive hydrocarbons was announced to obtain high-quality bio-fuels with high energy density, low freezing point and viscosity, which includes several reaction processes. A direct hydro processing pathway to produce reactive alkanes (C9–C15) should be more efficient and profitable. However, chemicals derived from natural oils remain unable to compete effectively with petrochemical industry products as a result of a huge amount of raw material and production costs (Figure 4). To improve the economic aspect of these processes, the choice of raw materials and reaction conditions, and the use of effective catalysts are crucial. TM/Z catalysts have potential due to the acidic nature of the surface and the ability to modify it with a wide range of TM, which opens up prospects for product composition management (Mbaraka and Shanks, 2006).

In (NOF) conversion processes acid catalysts are required for cracking, isomerization, alkylation, hydration, and dehydration. Zeolites are successfully used catalysts for NOF conversion due to its high stability, ease of separation and acidity that can be tuned by varying the ratio of Brønsted/Lewis acid sites, i.e., modifying the zeolite surface during acid-base pretreatment or ion exchange with TM cations. It is noteworthy that most of the NOF molecules are larger than the zeolite micropores. Recently, zeolites have been expanded in NOF conversion by introducing mesopores into the zeolite crystals. The mesoporous nature of the catalyst provided several advantages like: 1) rapid mass transfer, favorable adsorption, desorption and surface diffusion of reagents and products, 2) access of acid sites to bulky oligomer, 3) synergistic effect between acid sites in mesopores and micropores. These properties of the zeolites boost the catalytic activity, product selectivity, and catalyst life (Wang L. et al., 2017).

The best bi-functional zeolite-based catalysts for approaching the composition of oils to aviation fuel will be the ones containing transition metals that provide hydroprocessing which includes hydrodeoxygenation, hydrocracking, hydrogenation and hydroisomerization. Previous studies indicated that zeolite-supported noble metal catalysts have incomparable performance owing to the combining of superior hydrodeoxygenation ability of noble metals with the tunable acidity of zeolite supports (Snåre et al., 2006; Philippaerts et al., 2011; Sotelo-Boyás et al., 2011; Van Aelst et al., 2016; Yang and Carreon, 2017; Crawford and Carreon, 2018; Niu et al., 2020). This section considers the main processes of NOF and utilization by-product of the biodiesel industry, for which zeolite-based catalysts can be successfully used. However, it is hard to conclude the best catalyst since various experimental conditions were reported in the literature.

Among the base TM in the composition of TM/Z catalysts of natural oil hydrotreatment, the most significant attention in the literature is paid to nickel. Ni is a well-known catalyst for hydrotreatment, in addition, it has a strong capacity to cleave C-C bonds. The combination of the catalytic properties of Ni and zeolite opens up interesting prospects for biofuel production and is currently being actively studied (Niu et al., 2020). Ni NPs supported on hierarchical meso-Y (Cheng et al., 2019a), sulfonated meso-Y (Cheng et al., 2019b), and desilicated meso-Y (Zhang Z. et al., 2019) were applied for hydrocracking conversion of microalgal oil to jet fuel range hydrocarbons and the reaction parameters were optimized to achieve iso-alkanes. The effect of zeolite as support for the hydrotreatment of vegetable oil was studied by varying the Ni or loading on various zeolites (Wang et al., 2014). The 8 wt% Ni/SAPO-11 catalyst exhibited superior activity with high selectivity towards isomerized product due to moderate acidity and balanced meso-micropore channels. Diesel-range alkanes production was performed over Ni/zeolite from different feedstock for example, Ni/Hβ used for fatty acid methyl esters (Chen et al., 2014), Ni/micro-mesoporous β for methyl palmitate (Papanikolaou et al., 2020), Ni/β for vegetable oil (Wang et al., 2014), Ni/ZSM-5 for palmitic acid (Ojeda et al., 2018) and Ni/MOR for stearic acid (Crawford et al., 2020), etc. For hydrogenation of waste cooking oil into jet fuels, multicomponent Ni-contained zeolite-based materials like NiMo core-shell supported on hierarchical USY@Al-SBA-15 (Zhang et al., 2018) and NiCoMo oxides supported on an A-type zeolite (Asiedu and Kumar, 2019). The catalytic pyrolysis of the lipid-extracted residue of *Tribonema* with a maximum yield of alkylbenzene was reported over 6% NiO-2% MgO/ZSM-5 (Ji et al., 2017). The low-temperature selective cracking-dehydrogenation-aromatization of tree-borne oils to xylene-rich aromatics was performed by Zn/Y (Singh et al., 2020). Also Zn or Cr ion-exchanged with MFI was applied as a catalyst of rapeseed oil hydroconversion to aromatic hydrocarbons (Dedov et al., 2016).

Fatty acids are long chains aliphatic carboxylic acids naturally occurring in the form of triglycerides, cholesteryl esters and phospholipids. Apart from a dietary source, fatty acids and their esters are the primary sources for the soap, detergent, and lubricant industries. Therefore, utilization of nonedible fatty acids/oils for the production of valued products is more advantageous as it does not impact on human life (for example, increase of food cost). The bimetallic FeSn/BEA catalyst was used to convert microalgae residue into lactic acid under mild reaction conditions (Xia et al., 2021). Various metal ions like Mo, Co, Fe, Cr supported on HZSM-5 were used as catalysts for vegetable oils hydrolysis with different lipid compositions into polyunsaturated fatty acids (Robin et al., 2017).

#### Glycerol Valorization

**FIGURE 5:**
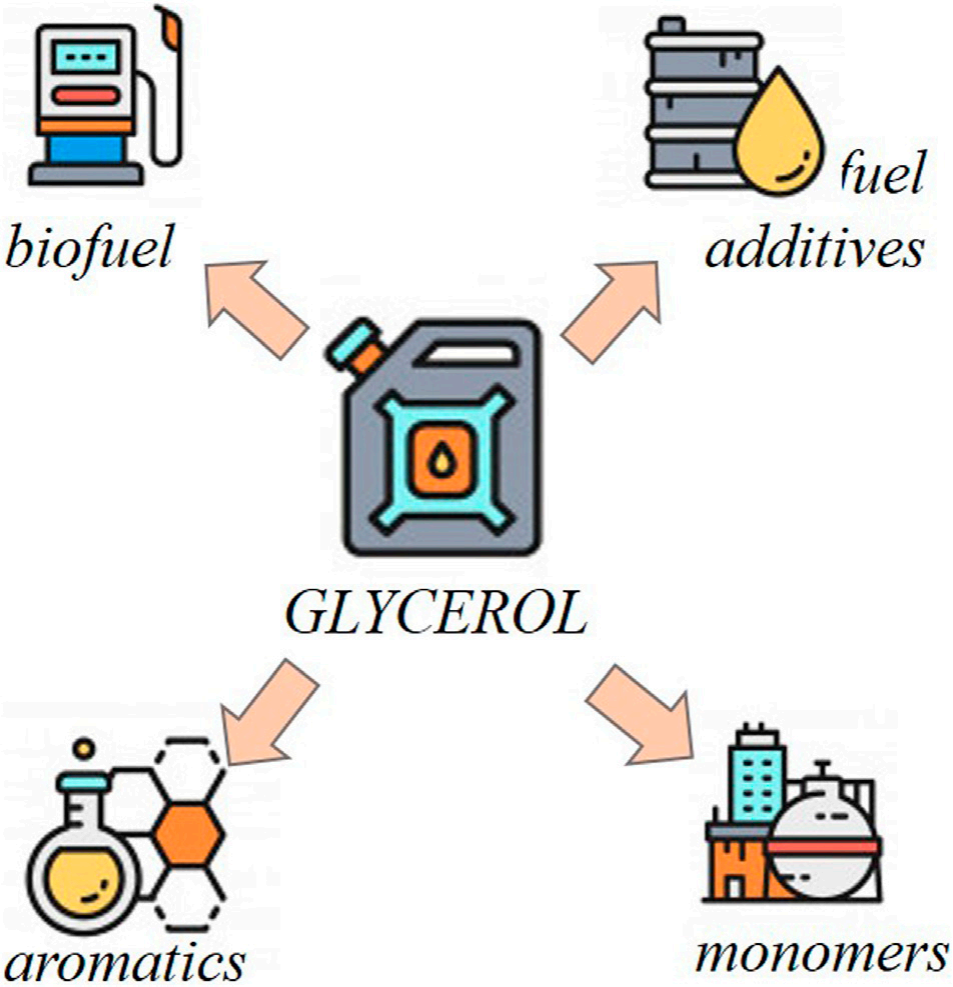
Glycerol transformation over TM/Z catalysts.

Glycerol is produced on a large scale as a byproduct in the biodiesel production industries. Several routes reported to transform glycerol, such as hydrogenation, dehydration, dehydrogenation, oxidation, aromatization, esterification, etc. Zeolite is the one of the catalysts reported for the transformation of glycerol into value-added products (Figure 5). Due to their mesoporosity, zeolite crystals improved their catalytic performance in glycerol transformation (Wang L. et al., 2017). Several valued products are derived from glycerol, however in this section we discuss the role of zeolite in the transformation of glycerol by oxy-dehydration, hydrogenolysis, aromatization techniques etc. Also, few reports on production of syngas and fuel additives from glycerol using zeolites are discussed.

Among biomass feedstock, glycerol has much attention, because it is a co-product during the triglyceride transesterification process to produce biodiesel. Oxy-dehydration of glycerol is one of the most interesting economic processes because it can produce acrolein, an important chemical used in various industries. The ZSM-5 gives high catalytic activity and outstanding selectivity in oxy-dehydration of glycerol but deactivates rapidly due to coke formation. However, the space velocities used in reported works are usually low and blockage of the pores by formation of carbonaceous deposits is favored. Recently, TM/Z have been investigated as potential catalysts for the oxidative dehydration of glycerol because of their acid and redox properties. Most studies of TM/Z catalysts in the literature suggest mixed metal (W, V, Mo) oxides, which presented the higher selectivity to acrylic acid.

Zeolites of different topologies: FAU, FER, MEL, MFI, MOR, MWW and OFF were synthesized and further impregnated with 5% vanadium and they showed high conversions of glycerol (100–78%). The acrolein selectivity decreased with the total density of acid sites in these zeolites. The selectivity of acrylic acid was related to the ability of each topology of stabilizing the redox pair V^5+^/V^4+^. The best performances were observed for zeolite catalysts with MWW, BEA, and MFI topologies (Silva et al., 2017). The main advantage of these catalysts was that they present similar acrylic acid selectivity operating under more drastic conditions than those employed for the catalysts based on mixed oxides, the main disadvantage is still the deactivation by coke. Mixed oxides of Mo_x_V_y_O_z_/H-ZSM-5 were applied for gas-phase glycerol dehydration-oxidation coupled reactions in which acrylic acid was the main product. This catalyst demonstrated only 6% of deactivation during 8 h under reaction conditions, while bulk mixed oxide and the pure ZSM-5 zeolite activities decreased by 20 and 31%, respectively (Possato et al., 2020).

Iron is an alternative active component for V which allows the activation of the catalyst by the reduction by glycerol of part of Fe(III) into Fe (II) species, which stabilizes glycerol conversion. The Fe/H-MCM-22 catalyst showed better performance compared to the Fe/zeolite and V/zeolite bi-functional catalysts (dos Santos et al., 2019). In the case of Fe_x_-BEA-Y, the Fe(III) species in the framework of zeolites was highlighted, moreover, the Fe(III) tetrahedral species are thought to be the main active redox sites for the formation of acrylic acid (Diallo et al., 2016). The production of allyl alcohol from glycerol on ZSM5-supported iron catalysts, modified by rubidium deposition was reported and differences in product distribution and catalyst performance were explained by the combined effects of iron loading, catalyst acidity and changes in porosity of the catalysts (Sánchez et al., 2016).

Recently, phosphorus was included in catalyst composition to modify the acidity of the zeolite. For acrolein production, the secondary hydroxyl group of glycerol needs to be removed first to from 3-hydroxypropanal (3-HPA) as an intermediate, which was later converted to acrolein by the second dehydration step. Brønsted acid sites typically catalyze the first step, and the strength of the Brønsted acid is influenced by the Si/Al ratio. By introducing another acid component, two advantages were obtained: enhancement of the Brønsted acid, which accelerated the first step and more available Lewis acid, which promoted the second reaction.

The hydrogen phosphates of transition metals (CuHPO_4_, Mo_1/3_HPO_4_, ZnHPO_4_, NiHPO_4_, MnHPO_4_) supported on meso-HZSM-5 hybrid catalysts generated an improvement of strong acid sites and acrolein yield. Thus, the yield of acrolein and the life time of catalysts increased by controlling the reaction pathways and reducing coke formation (Ren et al., 2019). The oxi-dehydration of glycerol was catalyzed by H_5_PV_2_Mo_10_O_40_ loaded on to the ZSM-5 zeolite with high selectivity to acrylic acid. *In-situ* IR analysis suggests that acrolein molecules adsorbed on H_5_PV_2_Mo_10_O_40_/ZSM-5 were converted into acrylic acid due to the inhibition of side-reactions such as polymerization and auto-condensation, which induced coke formation, compared with the other Mo and V-based oxides loaded on ZSM-5 zeolite (Suganuma et al., 2018). In the case of phosphotungstic acid (PTA) supported Y-zeolite the total acidity and pore size increased with a raise in loading up to 20 wt% and decreased beyond this limit. PTA/Y application showed a total conversion of 100% glycerol and 79% selectivity towards acrolein among all tested catalysts (Viswanadham et al., 2018).

Propanediol is a monomer for the production of polymers and can be produced from glycerol by the hydrogenolysis technique. The hydrogenolysis of glycerol yields products such as ethylene glycol and the valuable 1,2-propanediol (1,2-PDO), 1,3-propanediol (1,3-PDO), β-carotene, lactic acid, propionic acid, epichlorohydrin, ethanol, syngas, and H_2_, which are used in the manufacture of polymers, resins, functional fluids, foods and cosmetics, while also being important for the food and beverage industries. Hydrogenolysis of glycerol is generally performed over supported metal catalysts like Cu, Pt, Ru, Ir, Re, and Rh. Ruthenium has proved to be the most active catalyst to obtain 1,2-PDO although it promotes excessive C-C cleavage and the subsequent formation of undesired products, mainly methane. However, selectivity toward 1,2-PDO has been improved by adding acid co-catalysts such as TM/Z (Jin et al., 2014; Gallegos-Suarez et al., 2015). Limited reports are available on the selective formation of 1,3-PDO provided by the zeolite-supported Pt, Cu, and Ir (Priya et al., 2016a; Priya et al., 2016b; da Silva Ruy et al., 2020).

Since Ru and Pt are expensive, many researchers have studied and developed catalysts based on transition metals such as Ni, Cu, Co, Zn, which have lower cost and show promising results. Among transition metals, copper-based catalysts appear to be very efficient in the hydrogenolysis of glycerol due to their high activity in C-O bond cleavage. Highly dispersed copper oxide species supported on a Y-zeolite with different Si/Al ratios (from 5 to 60) were active in glycerol hydrogenolysis showing 92% conversion of glycerol and 83% selectivity to 1,2-PDO (Mitta et al., 2018). The dealumination of ultrastable Y-type zeolite resulted in good dispersion of Cu and selective molecular diffusion to 1,2-PDO (Niu et al., 2014).

Nickel-based catalysts promote hydrogen formation because of their high catalytic activity in the cleavage of C-C, O-H, C-H bonds of oxygenated organics, and also promote the removal of adsorbed carbon monoxide by the water-gas shift reaction. Since Ni and Cu have a different role in the process of glycerol hydrogenolysis without external H_2_ addition their combination with a zeolite support seems prospective. Bimetallic CuNi catalysts supported on hierarchically porous SAPO-11 zeolite showed extraordinary catalytic activity in hydrogenolysis of glycerol. The mesopores generated in M-SAPO-11 alleviate the diffusion barriers of reactants that are present in conventional SAPO-11 microporous channels during the liquid-phase reaction. Besides, the added Ni promotes the formation of a highly dispersed Cu active phase, which makes the bimetallic catalyst superior to the monometallic one. Also, the temperature has a more significant impact on glycerol conversion for various technological parameters (Li et al., 2019b). Again, the CuNi/Y catalysts also exhibited good catalytic stability (de Andrade et al., 2020).

Aromatization of glycerol is one of the promising routes to obtain aromatics, which would not only solve the problems of the crude oil conversion but also the transformation of the glycerol waste from the biodiesel industry into valid chemicals. Zeolite catalysts exhibited higher yield of aromatics than other catalysts, which can be explained by their synergetic acidity and mesoporosity. HZSM-5 has proved to be the most efficient catalyst for the production of aromatics due to its tunable acidity and three-dimensional, 10-ring micro pores matching the size of the aromatic molecules. However, bare HZSM-5 zeolite has poor activity and aromatics selectivity. Generally, the introduction of metal species in HZSM-5, such as Mo, Zn, Ag, Ni, Cu, Sn, either on the external surface or in the framework, is a frequently-used method to improve activity and aromatics yields during the aromatization reaction (Wang F. et al., 2017). Comparison of the catalytic properties of Sn incorporated hierarchical HZSM-5 and HZSM-5 in the glycerol aromatization showed that the remarkably improved performance in catalytic activity and stability observed for the H[Sn, Al]ZSM-5 catalyst can be attributed to the synergy between Sn species doped into HZSM-5 and the generated micro-mesoporous structure by alkali treatment (Yang et al., 2018). Zn species were introduced by the atomic layer deposition method in the form of thin-film into HZSM-5, where Sn incorporated into the framework improved the reusability of catalysts in glycerol aromatization. The synergistic effect of hybrid metal species and the interaction between metals and supports improved the performance in aromatization of glycerol (Wang F. et al., 2017).

Some reports are available on syngas production from glycerol, for which the most studied metal catalyst is Ni. The Linde-type 5A zeolite was used for Ni dispersion to produce synthesis gas (Huang et al., 2014). However, the strong interaction between the zeolite and Ni was modified with multi-metal ions (La, Ca, Mo), which reduced the acidity of the catalyst and enhance the glycerol conversion to syngas rather than to methane formation. Glycerol was used as a source for the production of olefins at high temperatures using Fe, Nb, and Mo containing HZSM-5 catalyst (Lima and Perez-Lopez, 2019). Among the studied metals Fe exhibited high selectivity towards propylene due to its relatively acidic strength at low temperatures higher than the Nb-ZSM-5. The latter case produced ethylene at high temperatures. As discussed in the above section, acrolein is formed due to the dehydration of glycerol over zeolite-based catalysts. The selective reduction (by hydrogen transfer) of acrolein to allyl alcohol was reported by Almeida et al. (2019) using vanadium supported beta zeolite, without adding of an external reductant.

The application of zeolites is not only limited to the glycerol valorization techniques, but also are used in the synthesis of bio-fuel additives (Solketal and t-butylethers of glycerol), olefins, glycerol carbonate, glycedol, glycerol oxidation, etc. For the acetalization of glycerol, among the reported various polyvalent metal ion incorporated on MOR catalysts, the synergistic effect of Cu and MOR made it the most effective catalyst for the production of solketal with high selectivity (Priya et al., 2017). In another report, V_2_O_5_ deposited in lamellar ferrierite and ITQ-6 zeolites were reported for the solketal synthesis from glycerol (Vieira et al., 2018). The high activity and selectivity achieved in the case of ITQ-6 is due to the presence of meso/micro pores and better diffusion compared to the ferrierite zeolite. One of the fuel additives with high octane numbers is the ether of glycerol, for which Y-zeolite is used as a support for phosphotungstic acid dispersion (Srinivas et al., 2014). The high selectivity obtained for mono-ether rather than bi-/tri-ethers products that the Y-zeolite pore might control. Another important product from glycerol is lactic acid and/or its esters obtained by oxidation of glycerol. Metals such as Au, Sn, CuO incorporated in zeolites (beta and USY) have been employed as catalysts for the single-step conversion of glycerol to methyl lactate (Lu et al., 2017; Zhou L. et al., 2019). The Au/Sn-USY catalyst exhibited high selectivity of methyl lactate due to high dispersion of Au to the Sn-Au interactions, where Sn contributed to the modification of acidic zeolite property (Vieira et al., 2018). Interestingly, Fe-MFI zeolites were used as catalysts for the gas phase oxidation of glycerol to dihydroxyacetone (an intermediate for lactic acid production) using molecular oxygen (Lari et al., 2015). The Fe-zeolite prepared by hydrothermal synthesis followed by steaming resulted in well-dispersed FeO_x_ species in the framework that displayed enhanced activity compared with the impregnated catalysts. Metal (Sn, Zn, Ag) impregnated Y-zeolites are more efficient than the parent one in the synthesis of glycerol carbonate using CO_2_ (Ozorio et al., 2015). Also, glycidol can be derived from glycerol carbonate or glycerol.

### H_2_ Production and Storage

In the last decades, technological research and development have been directed towards the implementation and production of clean energies to achieve zero-carbon emissions (Ni et al., 2007; Chou et al., 2013; Voldsund et al., 2016). It is estimated that hydrogen could largely replace fossil fuels (Midilli and Dincer, 2008). Thus, hydrogen is predictable as a clean fuel and represents a potential solution for energy storage. However, one of the main problems with the use and management of hydrogen is its low volumetric energy density, which, for instance, limits its implementation in fuel cells for use in the electronics and automotive industries. In general, hydrogen as an energy source represents a challenge to solve the problems related with its production, storage, transportation, distribution, and economic viability (Abe et al., 2019; Nazir et al., 2020). This section will discuss recent examples found in the literature related to the production and storage of hydrogen with TM/Zeolites.

It is well known that noble metal-based catalysts such as Ru, Pt, Rh, Pd, etc. exhibit the best catalytic performance of H_2_ production in different processes (Hu et al., 2019; Siang et al., 2020). However, the high costs of these metals limit their research and applications. In this sense, transition metals such as Co, Ni or Cu are the alternative for a new generation of catalysts to produce this fuel of interest (Luconi et al., 2019; Ogo and Sekine, 2020). In order to obtain a good dispersion and availability of the active sites, several zeolites must be explored to improve catalytic performance.

Currently, the primary sources of hydrogen production at an industrial scale is steam reforming of natural gas or light hydrocarbons (Cheekatamarla and Finnerty, 2006). However, other methods continue to be explored for production, such as photolysis, electrolysis, and thermal processes, etc. (Wan et al., 2019; Zhu et al., 2020a; Holade et al., 2020). Among the methods for hydrogen production with a great potential is the decomposition of methane, since it can contribute to the reduction of CO_2_, in addition to being a more energy-efficient process (endothermic). Metal catalysts for hydrogen production *via* decomposition of methane as Ni, Fe and Cu have achieved very promising results due to the high catalytic activity and stability in this process (CH_4_ conversions above 50% and H_2_ production above 60%) in the 600–900°C temperature range (Ashok et al., 2007; Ashik et al., 2015). In terms of TM/Z, for instance, Nasir Uddin et al. (2015) reported H_2_ production with Ni catalysts supported on zeolite Y through thermocatalytic descomposition (TCD) of methane. They showed that Ni/Y catalysts with a metal loading of 30 wt% achieved the highest hydrogen production at 650°C.

Other alternatives to produce H_2_ are compounds with -OH groups. Very recently, H_2_ production utilizing aqueous phase reforming (APR) with transition metals supported on zeolites from compounds derived from biomass showed a high potential. Gogoi et al. (2020) reported the catalytic activity of Ru exchanged on NaY zeolite in the APR using glycerol and ethylene glycol. The authors found that Ru/NaY catalysts with 3 wt% of the metal presented a glycerol conversion >80% and H_2_ selectivity >70%, associated with the suitable metal loading, good dispersion, high availability of Ru^0^ active sites and high activity for water gas shift reaction. They suggest that these catalysts have good potential for H_2_ production. According to Contreras et al. (2014) in their literature review about H_2_ production by ethanol steam reforming (ESR), the catalysts based on Ni, Co, Fe, and Cu supported in different matrices are the most studied and much cheaper than catalysts based on noble metals. Grzybek et al. (2020) reported Co-based catalysts supported on USY and ZSM-5 zeolites for ESR, in which both catalysts achieved a 100% ethanol conversion, meanwhile the Co/ZSM-5 catalyst exhibited high selectivity (75%). In any case, the selectivity to H_2_, CO_2_, and C_2_H_4_ depended on the ethanol-water molar ratio and the catalytic activity was associated with the reoxidation of Co^0^/Co^2+^ sites. In the case of nickel-based catalysts, the high activity to dissociate the C-C bond has generated various investigations. Lately, Wang et al. (2020) tested bioethanol steam reforming using a new kind of Ni core-shell catalysts supported on hierarchical beta zeolite. The conversion of ethanol and selectivity of hydrogen was higher than 85 and 70%, respectively. These results were associated with the role of morphology, Ni metal charge and its dispersion in the zeolite. Li et al. (2019c) tested a group of core-shell catalysts in the ESR, they prepared core-shell structures based on BEA and MFI zeolite crystals with Ni, Cu, and Co incorporated in several stages. All the catalysts showed a hydrogen selectivity greater than 50% associated with good metallic dispersion in the core-shell structures and the presence of Cu and Ni centers.

A possible solution for hydrogen storage are solid-state materials capable of efficiently capturing and releasing it. In this sense, aluminosilicates represent a viable low-cost alternative (Regli et al., 2005; Jhung et al., 2007; Chung, 2010; Kumar et al., 2020). The incorporation of transition metal cations can improve hydrogen adsorption capacity as they function as binding sites for hydrogen molecules (Kubas, 2007; Kozyra and Piskorz, 2016). Altiparmak et al. (2019) investigated the H_2_ storage capacity of Cu-exchanged ZSM-5 zeolites using adsorption calorimetry. According to H_2_ adsorption isotherms, Cu supported on mesoporous ZSM-5 sample with H_2_/Cu ratio of 1.03 achieved the highest H_2_ storage capacity around 0.03 wt% at 50 kPa, associated with Cu(I)-sites. Similar materials were studied by Ipek et al. (2018), and they reported that Cu-exchanged over SSZ-13 zeolites achieved H_2_ adsorption of 0.05 wt% at 1 atm.

### Theoretical Studies of Transition Metals on Zeolites

The use of zeolites as carriers on their surface or in the form of inclusions within their voids of atomically dispersed transition metals is of considerable interest in catalysis. Much attention to such systems is associated with the manifestation of synergistic effects between the applied metals’ components and the zeolite matrix. In this sense, the theoretical studies carried out in terms of calculations of first principles have played a significant role since they have provided important information on the electronic structure of different zeolitic frameworks and various transition metals deposited in the channels. In general, catalytic performance of zeolites is characterized theoretically by evaluating the activation barriers and the reaction energies of the heterometallic sites in the presence of the molecule of interest. Compared to *ab-initio* methods, the scaling/precision cost of studies based on the density functional theory (DFT) method are considerably lower (Nicholas, 1997). However, the correct prediction of the catalytic performance of zeolites depend on the type of pseudopotential used to minimize errors (Grecu et al., 2018; Wang, 2018).

**FIGURE 6:**
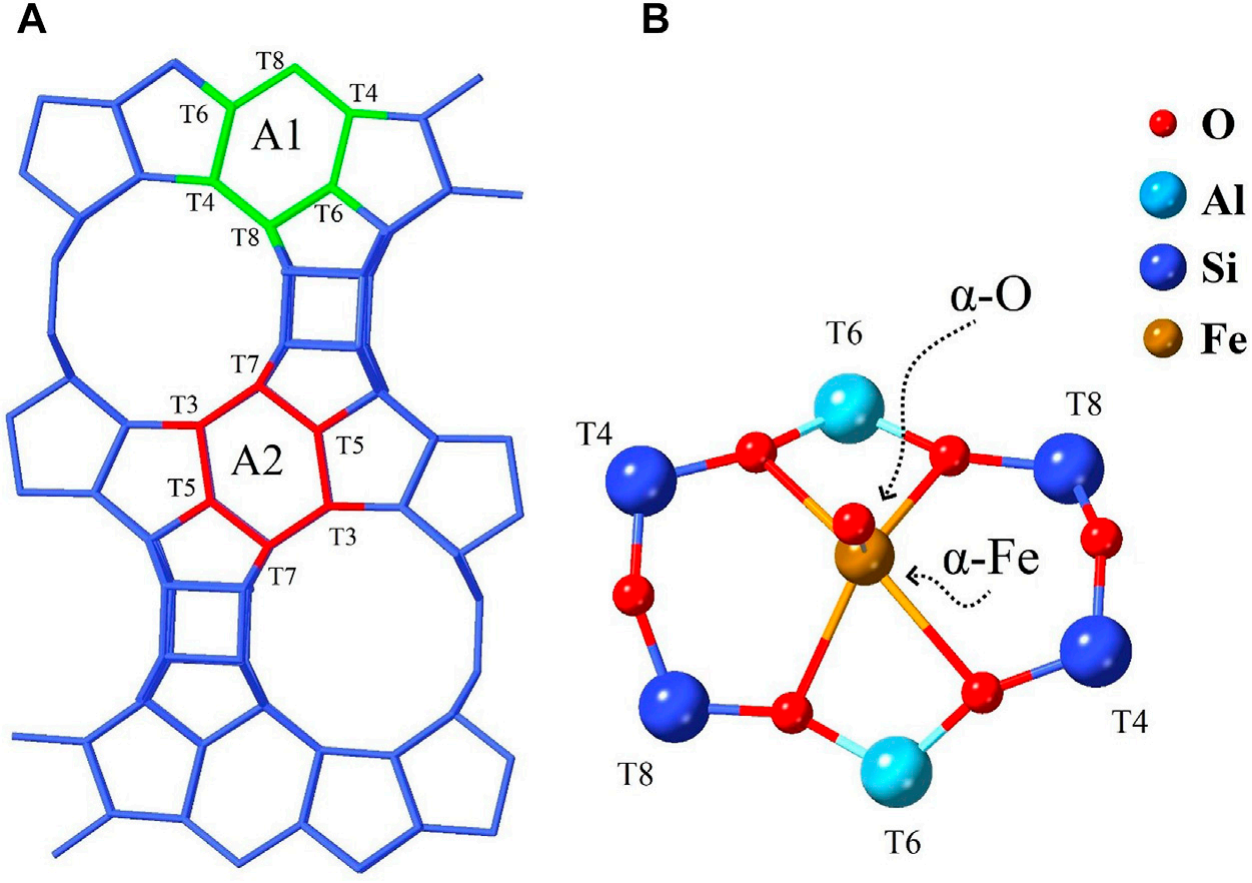
**(A)** Sketch of the BEA zeolite highlighting two six-membered rings (6MR), designated as A1 and A2, and **(B)** the energetically most favorable configuration of the active site α-Fe(II) and its intermediate α-O in the A1 ring. In addition, the various tetrahedral units are marked with a capital T followed by a number. The same numeric label represents tetrahedral units that satisfy the same symmetry rules. Reprinted with permission from Snyder et al. (2016). Copyright 2016 Nature.

Due to the recognized impact that zeolites have on petroleum refining, these are expected to have a potential application in the direct conversion of MTM (Vermeiren and Gilson, 2009; Choudary and Newalkar, 2011; Klerk, 2018). Thus, although zeolites are the subject of many theoretical investigations, the study of the inclusion of transition metals in zeolites has been mainly aimed at understanding the nature of catalytic processes in this field. In this direction, one of the most outstanding works was that by Pannov et al. (1990), who reported experimentally that iron loading in H-ZSM-5 zeolite could efficiently decompose N_2_O at low temperatures (below 300°C). As a further effect of this decomposition, highly reactive oxygen bonded to the zeolite surface (termed α-O) could be involved in the oxidation of CH_4_ at room temperature. It would not take long to confirm the selective oxidation of methane to methanol in the Fe-ZSM-5 zeolite (Sobolev et al., 1995). In addition to Fe-ZSM-5 zeolite, N_2_O decomposition has also been observed for zeolites such as Fe-FER, Fe-BEA, and Fe-MOR (Centi et al., 2004; Jíša et al., 2009). Of this group, the Fe-BEA zeolite presents the highest intensity of the ligand-field bands, a fact from which Snyder et al. (2016) took advantage to investigate the nature of the active site [termed α-Fe(II)]. In particular, the results of magnetic circular dichroism (MCD) revealed that the nature of the active site α-Fe(II) is mononuclear (see Figure 6), high-spin, and presents coordination with square-planar geometry. Similarly, the reactive intermediate α-O showed a mononuclear nature and high-spin species Fe (IV)=O. Besides, their studies based on DFT (under cluster approach) suggest that this environment is stable and viable only in a very particular six-membered ring (6MR) configuration.

**FIGURE 7:**
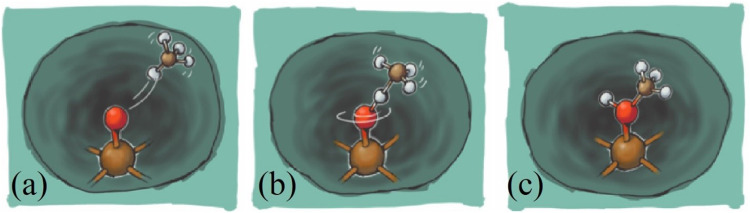
Illustration of the rebound mechanism for methane to methanol conversion. **(A)** CH_4_ molecule in the vicinity of the Fe-oxo site, **(B)** adsorption of methane and detachment of one of the H’s by the a-O intermediate, and **(C)** the reconfiguration of both H and a-O. The latter configuration decreases the strength of the a-O and a-Fe bond and, by the effect of thermal perturbations or momentum transfer from molecules present in the flow, can be desorbed, resulting in the desired product. At the end, the active site is available to be occupied by a new a-O and as a consequence, the catalyst is regenerated. Reprinted with permission from Göltl et al. (2016). Copyright 2016 American Chemical Society.

For other zeolites, it is challenging to determine experimentally the nature of the active site and the factors that influence its reactivity due to the presence of the inactive spectator iron species. However, for the ZSM-5 zeolite, it has been shown that in addition to the monovalent iron oxide cation [FeO]^+^ (Yoshizawa et al., 2000), other cations such as the divalent iron oxide cation [FeO]^2+^ (Yoshizawa, 2006; Rosa et al., 2010), and dinuclear iron complexes (Yoshizawa and Yumura, 2003) are stable and show potential applications for MTM conversion. Mahyuddin et al. (2016) studied the inclusion of different monometals [MO]^+^ (M=Fe, Co, Ni, Cu) in the zeolite ZSM-5 for the conversion of MTM, predicting that the reactivity toward C-H bond dissociation presents the following trend [CoO]^+^-ZSM-5 < [NiO]^+^-ZSM-5 < [FeO]^+^-ZSM-5 < [CuO]^+^-ZSM-5. In contrast, the trend for methanol selectivity increases according to [FeO]+-ZSM-5 < [CoO]^+^-ZSM-5 < [NiO]^+^-ZSM-5 < [CuO]^+^-ZSM-5. In this work, the catalytic cycle that arises for the MTM conversion is similar to the “rebound mechanism” (see Figure 7) proposed for C-H bond activation by non-heme Fe(IV) complexes (Shaik et al., 2004; Cho et al., 2012; Göltl et al., 2016).

Interestingly some Cu-exchanged zeolites (Groothaert et al., 2005; Alayon et al., 2012; Narsimhan et al., 2016; Le et al., 2017) have shown that the precursor can be activated with molecular O_2_, which is less expensive than N_2_O. For Cu-exchanged Na-ZSM-5 (Cu-Na-ZSM-5), theoretical-experimental results showed that the active site is mono(μ-oxo)dicopper species [also referred to as (Cu_2_O)^2+^] (Woertink et al., 2009), the same active site is found for Cu-Na-ZSM-5 (Vanelderen et al., 2015). According to Sushkevich et al. (2017), this step can be excluded if the reaction is conducted in the presence of water (or anaerobic condition), which acts as a source of oxygen atoms. Theoretical results (Palagin et al., 2019) show that the reaction also occurs in a similar way to the rebound mechanism, but the oxygen provided by the water molecules reoxidizes the active centers of copper, re-stabilizing them to facilitate the desorption of the product (methanol). Due to the broad potential that Cu-exchanged zeolites have for MTM conversion, other active sites such as monocopper [Cu(II)OH]^+^ species (Kulkarni et al., 2016; Ipek et al., 2017) and dicopper peroxo [Cu2O2]^2+^ species (Ipek et al., 2017; Pappas et al., 2017; Oord et al., 2018) have also been proposed. In addition to the conversion of MTM, there are other exciting types of catalytic reactions where transition metals have been studied. For instance, ZSM-5 has been loaded with cations of Au, Be, Co, Cu, Mg, and Zn to study the conversion of CH_4_ and CO_2_ into acetic acid (Panjan et al., 2012; Gerceker et al., 2017) or for the dehydrogenation of ethanol to acetaldehyde (Maihom et al., 2014). The methane conversion to ethylene and aromatics has been evaluated in ZSM-5 when are loaded with Pt and PtSn nanoparticles (Gerceker et al., 2017). Also, the adsorption of H_2_S has been studied by including cations of different metals (Fe, Co, Ni, Cu, and Zn) in ZSM-12 (Fellah, 2016). Recently, the migration of Cu^+^ has been studied as an effect of introducing cations of Mg^2+^, Ca^2+^, Sr^2+^, Ba^2+^, and La (OH)^2+^ species into Y-zeolite for the synthesis of dimethyl carbonate (Zheng et al., 2020).

## Summary and General Conclusion

Current challenges in the field of environmental protection and sustainable energy production are inspiring the development of catalytic processes both for direct removal of pollutants and for cleaner and less energy-intensive production. The existing focus is on the development and implementation of processes inspired by the growing understanding that humanity must turn hazardous waste into valuable products, combining the removal of pollutants with the production of vital materials. Among the many catalysts, TM/Z materials based on transition metals supported on zeolite supports have attracted active attention.

The combination of such active substances as ions and nanoparticles of transition metals, and such a nontrivial carrier as zeolites in one material makes it possible to realize synergistic effects created by the combination of their intrinsic properties. Transition metals have long been known as useful catalysts to cleave C-C, C-H, C-S, C-O, C-N and C-Metal bonds. The well-known specific properties of zeolites are useful to create highly dispersed active centers within their crystallographically determined porosity. The result is a new generation of TM/Z based catalysts with improved physicochemical properties. They are capable of activating and converting methane, carbon dioxide, plastic waste, biomass, etc. into useful raw materials.

Capturing CO_2_ by zeolites and generating added value through converting it to chemicals is a very good alternative to reduce atmospheric emissions. The steric properties of zeolite matrices may be responsible for the selectivity towards isoparaffins and products exceeding C_5_ in TM/Z bifunctional catalysts.

The emerging alternative to converting methane into products with higher added value through a partial oxidation process involves reducing its emissions into the atmosphere. Various TM/Z were tested and the multicomponent catalysts showed the best performance in the catalytic reaction. It is believed that zeolite acidity is a key factor in the methanol adsorption/desorption process, allowing methanol to be desorbed before it is completely oxidized to CO_2_; therefore, modification of the zeolite matrices is an important task.

Recent theoretical studies have provided a deeper understanding of the synergistic role of interactions between the various components and the zeolite matrix. The retention of metal clusters and/or cations within a zeolite depends on its very specific crystal structure and its chemical composition (e.g. Si/Al ratio). The role of the spatial distribution of trivalent metals (among which Al is the best known) such as Fe, Ga, Y, etc. in the tetrahedra of the zeolite framework continues to be a topical issue of research in the field of catalysis. The future of custom catalyst development and catalyst tuning depends on a deeper understanding of all of these factors. Furthermore, research based on the use of artificial intelligence and machine learning may become widely used methodologies for optimizing the synthesis conditions of the most active, selective and stable TM/Z catalysts based on an extensive database of experimental studies.

Municipal solid waste can be an important source of hydrocarbons as it contains plastics, plastic containers, food packaging materials, paper, rubber, textiles and non-recyclable packaging materials, as well as biomass (food waste) and other solid waste from various sources. Plastics may be very convenient for a variety of uses, but as a secondary result, a lot of plastics end up in household wastes. The chemical structure of most plastics makes them resistant to many natural degradation processes; thus, they degrade slowly. Together, these two factors allow large volumes of plastic to end up in the environment as misplaced waste and to remain in the ecosystem.

Methods are being developed for converting waste to energy or converting waste into a fuel source. Most processes produce combustible fuels such as methane, methanol, ethanol, or synthetic fuel. TM/Z are successfully used as typical catalysts for the decomposition of polymer waste. Recent studies have shown the importance of such catalysts, as well as the noble metals supported on zeolite, for converting biomass to jet fuel due to their superior hydrodeoxygenation capacity and controlled acidity of zeolite carriers. In addition, zeolites with transition metal species have turned out to be promising catalysts in the production of fuel additives from glycerin. There are several ways to convert glycerol using TM/Z catalysts. Hydrogenolysis of glycerol leads to the formation of several valuable products, including Ni-based catalysts successfully providing hydrogen formation.

Among the new environmentally friendly clean energy sources, hydrogen is the most interesting. The production of H_2_ by reforming on TM/Z catalysts using biomass-derived compounds is becoming an alternative to methane decomposition or steam reforming of natural gas. Typically, TM/Z catalysts have high conversions (>80%) and good H_2_ selectivity (>70%), which are associated with factors such as suitable metal loading, good dispersion, high availability of active sites, and high activity. Also, a number of studies have been carried out on the storage of H_2_ on zeolites with transition metal cations. These hybrid materials have demonstrated the ability to adsorb hydrogen, with TM cations functioning as binding sites for hydrogen molecules.

Finally, no matter how clean the fuel is, nitrogen oxides are inevitably formed during combustion. Recent advances in the removal of NO_x_ emissions from exhaust gases using TM/Z catalysts are based on the use of multimetallic materials. Through their use it is possible to expand the range of operating temperatures, which is usually 300–450°C. Research is underway to optimize the synthesis parameters and the optimal ratio of active phases. Parameters such as the order of component deposition, metal loading and metal deposition method strongly influence the final performance of multimetallic catalysts.

## Footnotes

1 Despite the generally accepted designation of zeolite structures in the form of a three-letter code (a list of zeolites can be found on the IZA website: http://www.iza-structure.org/databases/), in our article the names are given in accordance with how they were used by the authors of the original articles. Therefore, along with the systematic designations (such as MFI or FAU), traditional designations for the same structures (ZSM-5 or Y-zeolite) coexist in the text.
